# Identification and characterisation of isoprene‐degrading bacteria in an estuarine environment

**DOI:** 10.1111/1462-2920.13842

**Published:** 2017-07-21

**Authors:** Antonia Johnston, Andrew T. Crombie, Myriam El Khawand, Leanne Sims, Gregg M. Whited, Terry J. McGenity, J. Colin Murrell

**Affiliations:** ^1^ School of Environmental Science University of East Anglia, Norwich Research Park Norwich NR4 7TJ UK; ^2^ DuPont Industrial Biosciences 925 Page Mill Road, Palo Alto CA 94304 USA; ^3^ School of Biological Sciences, University of Essex Wivenhoe Park, Colchester CO4 3SQ, UK; ^4^ Earth and Life Systems Alliance Norwich Research Park, Norwich UK

## Abstract

Approximately one‐third of volatile organic compounds (VOCs) emitted to the atmosphere consists of isoprene, originating from the terrestrial and marine biosphere, with a profound effect on atmospheric chemistry. However, isoprene provides an abundant and largely unexplored source of carbon and energy for microbes. The potential for isoprene degradation in marine and estuarine samples from the Colne Estuary, UK, was investigated using DNA‐Stable Isotope Probing (DNA‐SIP). Analysis at two timepoints showed the development of communities dominated by Actinobacteria including members of the genera *Mycobacterium*, *Rhodococcus*, *Microbacterium* and *Gordonia*. Representative isolates, capable of growth on isoprene as sole carbon and energy source, were obtained from marine and estuarine locations, and isoprene‐degrading strains of *Gordonia* and *Mycobacterium* were characterised physiologically and their genomes were sequenced. Genes predicted to be required for isoprene metabolism, including four‐component isoprene monooxygenases (IsoMO), were identified and compared with previously characterised examples. Transcriptional and activity assays of strains growing on isoprene or alternative carbon sources showed that growth on isoprene is an inducible trait requiring a specific IsoMO. This study is the first to identify active isoprene degraders in estuarine and marine environments using DNA‐SIP and to characterise marine isoprene‐degrading bacteria at the physiological and molecular level.

## Introduction

Isoprene (2‐methyl‐1,3‐butadiene) is emitted to the atmosphere, mainly by terrestrial vegetation, at a rate of approximately 550 Tg year^−1^, which is similar in magnitude to emissions of methane (Guenther *et al*., 2012; Kirschke *et al*., 2013). Isoprene undergoes rapid photochemical oxidation in the atmosphere, initiated by hydroxyl radicals, ozone (O_3_), nitric oxide, and nitrate and halogen radicals (Atkinson and Arey, 2003), resulting in the formation of low‐level ozone, a pollutant and global warming gas, and influencing the atmospheric concentration of OH radicals, which in turn alters the oxidation rate of the potent greenhouse gas, methane (Pacifico *et al*., 2012). The oxidation products form secondary organic aerosols (SOA) and cloud condensation nuclei, resulting in atmospheric haze and affecting the planetary albedo (Carlton *et al*., 2009).

Isoprene, which in deciduous forests can account for 80% of all hydrocarbons released (Lamb *et al*., 1987), protects plants against heat stress and reactive oxygen species (Sharkey *et al*., 2008; Zeinali *et al*., 2016). In the marine environment, micro‐ and macroalgae are the major producers of isoprene, although “bottom‐up” and “top‐down” estimates vary by two orders of magnitude between about 0.1 and 12 Tg year^−1^ (Palmer and Shaw, 2005; Luo and Yu, 2010; Shaw *et al*., 2010; Dani *et al*., 2017). Despite their relatively small contribution to the total global source, marine emissions may play a disproportionate and perhaps significant role in SOA formation over the oceans (Carlton *et al*., 2009; Gantt *et al*., 2009; Hu *et al*., 2013) with corresponding importance for global climate. Studies have reported isoprene concentrations in surface seawater in the 1–400 pM range (Bonsang *et al*., 1992; Milne *et al*., 1995; Broadgate *et al*., 1997; Baker *et al*., 2000; Matsunaga *et al*., 2002; Broadgate *et al*., 2004; Acuña Alvarez *et al*., 2009; Booge *et al*., 2016).

The only isoprene sink studied in detail is photochemical degradation. However, microbes in soils readily degrade isoprene (Cleveland and Yavitt, 1998; Gray *et al*., 2015) and isoprene‐degrading bacterial strains have been isolated, although most were not characterised in detail (van Ginkel *et al*., 1987a, 1987b; Ewers *et al*., 1990; Cleveland and Yavitt, 1997; van Hylckama Vlieg *et al*., 1998). Recently, however, El Khawand et al. (2016) showed that isoprene‐degrading *Rhodococcus* spp. were abundant in isoprene‐enriched soil microcosms and that members of the Comamonadaceae were also active in isoprene degradation. These authors developed PCR primers targeting *isoA*, encoding the alpha‐subunit of the oxygenase component of isoprene monooxygenase (IsoMO), which were used to retrieve *isoA* sequences from diverse environmental samples and provided a snapshot of the active terrestrial isoprene‐degrading community.

In the marine environment, Acuña Alvarez et al. (2009) investigated isoprene degradation at sites in the UK, France, and Indonesia and reported that samples from the Colne Estuary (UK) degraded isoprene in microcosms. The enriched samples were dominated by Actinobacteria, and several isoprene‐degrading strains were isolated (Acuña Alvarez *et al*., 2009). Mixtures of these bacteria consumed isoprene produced in microcosms by algal cultures, suggesting that isoprene degradation by bacteria could occur in these environments. Despite this potential, no studies have directly identified the metabolically active isoprene degraders in marine or estuarine environments or searched for the genes involved.

Isoprene metabolism is best characterised in *Rhodococcus* sp. AD45 (van Hylckama Vlieg *et al*., 1998; van Hylckama Vlieg *et al*., 1999; van Hylckama Vlieg *et al*., 2000; Crombie *et al*., 2015), originally isolated from freshwater sediment, which can grow on isoprene as sole carbon and energy source. The genome includes genes for a multicomponent IsoMO essential for isoprene metabolism (Crombie *et al*., 2015), most similar to other soluble diiron centre monooxygenases (SDIMOs) such as alkene monooxygenase from the propene (propylene) degrader *Xanthobacte*r *autotrophicus* Py2 (Small and Ensign, 1997). IsoMO oxidises the methyl‐substituted double bond of isoprene, yielding 1,2‐epoxy‐2‐methyl‐3‐butene (van Hylckama Vlieg *et al*., 2000). The epoxide ring is cleaved by conjugation with glutathione and catalysed by glutathione‐*S*‐transferase (IsoI). A dehydrogenase, IsoH, then oxidises the alcohol moiety of 1‐hydroxy‐2‐glutathionyl‐2‐methyl‐3‐butene to the carboxylic acid (van Hylckama Vlieg *et al*., 1999). The genes responsible form part of a cluster of 22, all of which were implicated in isoprene metabolism (Crombie *et al*., 2015).

The effect of release of isoprene to the atmosphere cannot be accurately predicted without understanding the cycling of isoprene in terrestrial and marine environments, necessitating detailed study of isoprene biodegradation. Here, we used DNA stable‐isotope probing (Dumont and Murrell, 2005) to identify active isoprene degraders in estuarine samples and isolated isoprene‐degrading strains, which were characterised at the physiological and genetic levels, thus expanding the diversity of known isoprene‐degrading microbes.

## Results

### DNA stable‐isotope probing of Colne Estuary samples using ^13^C‐labelled isoprene

**Figure 1:**
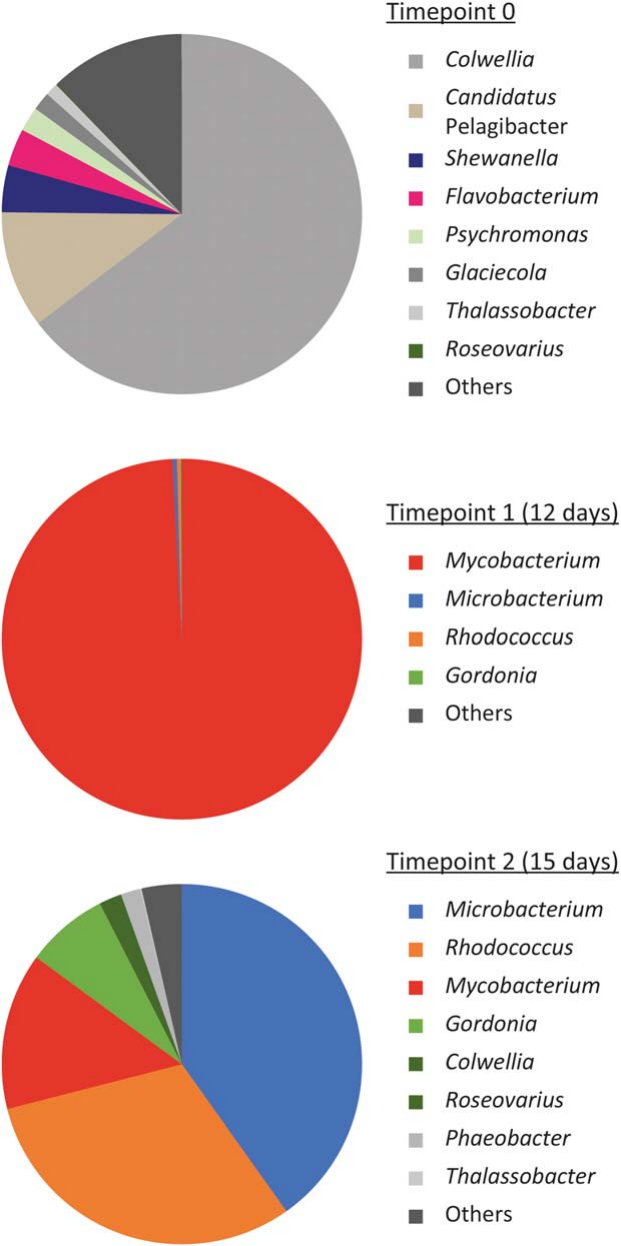
Bacterial community composition of DNA‐SIP isoprene enrichments. The unenriched timepoint zero community is shown together with the labelled communities at timepoints one and two (12 and 15 days), retrieved from the heavy fractions of ^13^C‐isoprene enrichments. The data show the mean of two replicates, except timepoint zero (one sample). All genera present with a relative abundance > 1% at any timepoint are shown.

Water and sediment samples were taken from the Colne Estuary (Wivenhoe, Essex, UK) and the bacterial community characterised by sequencing of 16S rRNA gene amplicons. The community was dominated (75%) by Gammaproteobacteria, principally *Colwellia* (65%) (Fig. 1). Also, abundant were *Candidatus* Pelagibacter (Alphaproteobacteria) (11%), with a lesser contribution from the Bacteroidetes genera *Flavobacterium* and *Aquimarina* (4%). We used DNA stable‐isotope probing (DNA‐SIP) to identify the active isoprene degraders. Samples were incubated with ^13^C‐labelled isoprene or ^12^C (unlabelled) isoprene (headspace concentration approximately 0.2%, vol/vol, corresponding to an aqueous phase concentration of approximately 26 µM) and sacrificed at two timepoints (12 and 15 days) when substrate carbon consumption was approximately 30 and 60 µmol g^−1^ (sediment). Following separation of ^13^C‐labelled and ^12^C (unlabelled) DNA by ultracentrifugation and fractionation, the active isoprene degraders were identified by sequencing 16S rRNA gene amplicons from the labelled and unlabelled (‘heavy’ and ‘light’) fractions of ^13^C‐isoprene incubations and the corresponding fractions of control ^12^C incubations. At 12 days, the 16S rRNA gene amplicons retrieved from the heavy DNA fractions were comprised almost entirely of sequences from *Mycobacterium* spp., which formed 99% of the labelled community (Fig. 1). These sequences were highly enriched (over 300‐fold) in the labelled (heavy) fractions compared to unlabelled (light) fractions from incubations with ^13^C isoprene but were found in the light fraction of ^12^C incubations and were not detected in the heavy fraction (Supporting Information Fig. S1), demonstrating assimilation of carbon from labelled isoprene into DNA. Most (93%) of these sequences were related to the 16S rRNA gene of *Mycobacterium rhodesiae* and 5% to the 16S rRNA gene of *Mycobacterium neoaurum. Microbacterium* spp. were also labelled, although they comprised a minor component of the active isoprene‐degrading community (0.4%). In addition to the 16S rRNA gene data, *isoA* amplicons from the labelled DNA at day 12 were generated using *isoA*‐specific primers (El Khawand *et al*., 2016). Two OTUs comprised 95% of these *isoA* sequences, the most abundant (91%) identical to *isoA* from isoprene‐degrading isolates obtained in this study, *Mycobacterium* sp. i61a, *Loktanella* sp. 8bn and *Gordonia* sp. i37 (described later), and the second most abundant (4.5%) related to *isoA* from *Rhodococcus* sp. AD45 (van Hylckama Vlieg *et al*., 2000) (90% nucleotide identity; Supporting Information Fig. S2).

At 15 days, 16S rRNA gene analysis revealed a more diverse community, with *Mycobacterium* (11–16% of rRNA gene sequences) now outnumbered by *Microbacterium* (36–43%, principally related to *M. oxydans*) (Fig. 1). *Gordonia* and *Rhodococcus* 16S rRNA genes were also abundant (7% and 31% of labelled DNA, respectively), but as these sequences were also present in light fractions of the ^13^C incubations in one replicate (Supporting Information Fig. S1), the data do not allow definite identification of these sequences as labelled. No representatives of phyla other than Actinobacteria were highly enriched in labelled fractions. To further investigate the physiology and regulation of the estuarine actinobacterial isoprene degraders, we set out to characterise representative isolates.

### Enrichment and isolation of isoprene‐degrading bacteria

**Figure 2:**
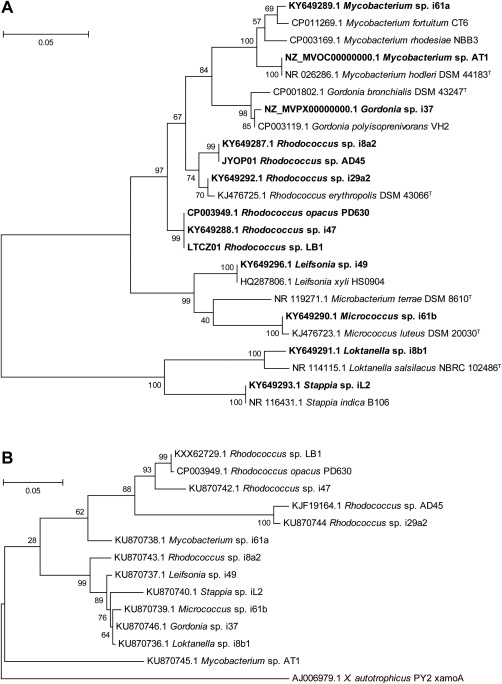
A. Phylogeny, based on 16S rRNA gene sequences, of known isoprene‐degraders (shown in bold), together with closely related non‐isoprene‐degrading strains. The tree was constructed in MEGA6 (Tamura *et al*., 2013) using the Neighbour‐joining method. All positions containing gaps and missing data were eliminated, and there were a total of 535 positions in the final dataset. B. Phylogenetic relationship of known isoprene‐degrading strains based on *isoA* sequences, together with alkene monooxygenase (*xamoA*) from *Xanthobacter autotrophicus* Py2 (Zhou *et al*., 1999). The tree was drawn in MEGA6 using the Maximum Likelihood method based on an alignment of *isoA* sequences. All positions containing gaps and missing data were eliminated and there were a total of 1010 nt in the final dataset. Scale bars indicate nucleotide substitutions per site. Bootstrap values (1000 replications) are shown at the nodes.

Water and sediment samples were taken from four locations to provide a broad selection of coastal, estuarine, and marine environments: Wivenhoe and Hythe (both on the Colne estuary, UK), Penarth, South Wales, UK, and the Western Channel Observatory L4 sampling station, located approximately 50 miles off the coast of Plymouth, UK. Enrichment cultures completely degraded isoprene added to the headspace (0.5%, vol/vol) within 4–5 days (data not shown). Several novel isoprene‐degrading isolates were obtained from these enrichments (Table S1) and together with isolates obtained previously (Acuña Alvarez *et al*., 2009) identified by 16S rRNA gene sequencing and their isoprene‐growth characteristics investigated. Table S1 shows the taxonomic affiliations, growth rates, and culture densities obtained during growth on isoprene as sole carbon and energy source. All of these isolates grew optimally at 0.5–3% (wt/vol) NaCl (with the exception of *Loktanella* sp. i8b1, which grew well in the range 0–4%, wt/vol) indicating that these were genuine marine/estuarine‐adapted strains (Supporting Information Fig. S3 and data not shown). IsoMO oxygenase alpha subunit (*isoA*) gene sequences were retrieved from all the isolates using gene‐specific primers (El Khawand *et al*., 2016), suggesting that they use a SDIMO to grow on isoprene as previously described (van Hylckama Vlieg *et al*., 2000; Crombie *et al*., 2015). Interestingly, the *isoA* sequences do not group congruently with their 16S rRNA gene‐derived phylogeny. The *isoA* sequences from highly diverse strains including both Gram‐positive Actinobacteria and Gram‐negative members of the Alphaproteobacteria form a group of near‐identical sequences (Fig. 2). Two isolates, *Gordonia* sp. i37 and *Mycobacterium* sp. AT1, were selected for further study since they were likely representative of isoprene degraders from this environment (based both on SIP data presented above and previous work; Acuña Alvarez *et al*., 2009), were capable of rapid and robust growth on isoprene and possessed relatively dissimilar *isoA* genes.

### Genomes of *Gordonia* sp. i37 and *Mycobacterium* sp. AT1

**Figure 3:**
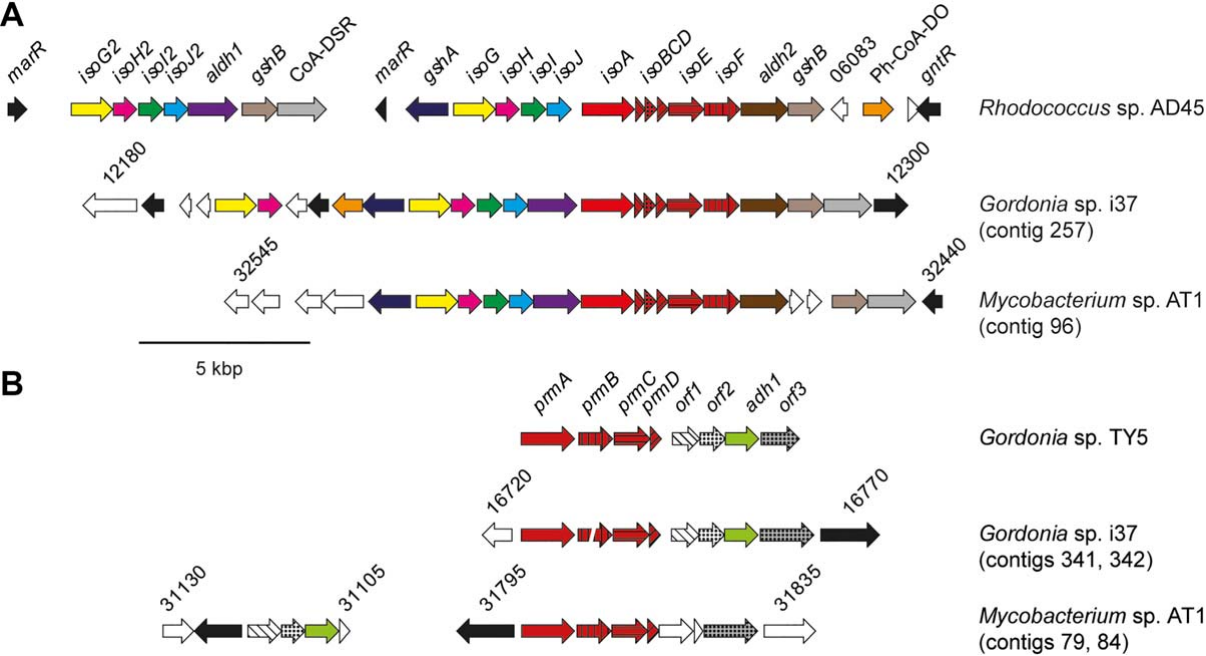
A. Arrangement of the isoprene gene clusters in *Rhodococcus* sp. AD45 (Crombie *et al*., 2015), *Gordonia* sp. i37 and *Mycobacterium* sp. AT1. B. The propane monooxygenase and associated genes in *Gordonia* sp. TY5, *Gordonia* sp. i37 and *Mycobacterium* sp. AT1. Open reading frames are coloured according to their homologues in *Rhodococcus* sp. AD45 (A) or *Gordonia* sp. TY5 (B). Hypothetical proteins and those with no close homologues adjacent to the gene clusters in *Rhodococcus* sp. AD45 or *Gordonia* sp. TY5 are shown in white. Regulatory genes are in black. CoA‐DSR, CoA disulfide reductase; Ph‐CoA‐DO, phytanoyl‐CoA dioxygenase.

To investigate the genetic basis of isoprene metabolism in these isolates, we generated draft genome sequences of *Gordonia* sp. i37 and *Mycobacterium* sp. AT1, as summarized in Table S2. The genomes were screened for genes predicted to encode enzymes known to be capable of gaseous alkane or alkene degradation. Two SDIMOs were found in the genomes of each strain, but no other genes predicted to encode enzymes of gaseous alkane or alkene oxidation were identified, although putative cytochromes *cyp153* and *alkB* sequences similar to enzymes with C_5_–C_12_‐oxidising ability in other Actinobacteria were identified (Table S3) (Nie *et al*., 2014). One SDIMO from each strain showed high similarity to IsoMO from *Rhodococcus* sp. AD45 (Crombie *et al*., 2015), and a complete predicted isoprene metabolic gene cluster was identified in the genome of each isolate (Fig. 3A and Supporting Information Table S4). In comparison with each other, these clusters share a very similar layout. The structural monooxygenase genes *isoA‐F* are flanked by genes encoding two putative aldehyde dehydrogenases (*aldh1* and *aldh2*). Upstream (5′), on the same strand, *isoGHIJ* encodes a predicted racemase of unknown function, a dehydrogenase, and two glutathione‐*S*‐transferases. In *Rhodococcus* sp. AD45, IsoI catalyses the conjugation of isoprene epoxide with glutathione and IsoH is responsible for the two subsequent oxidation reactions, while no known function has been assigned to IsoG or IsoJ (van Hylckama Vlieg *et al*., 1999). In *Rhodococcus* sp. AD45, *isoGHIJ* is duplicated (Crombie *et al*., 2015) and copies of *isoG* and *isoH* (only) are upstream in *Gordonia* sp. i37, although not in *Mycobacterium* sp. AT1. These four genes and the genes encoding the monooxygenase are flanked, on opposing strands, by *gshA* and *gshB*, predicted to encode glutathione biosynthesis enzymes glutamate‐cysteine ligase and glutathione synthetase. The putative isoprene metabolic enzymes share 55–87% amino acid identity between the strains (Table S4). In terms of gene layout, the principal difference is that in *Mycobacterium* sp. AT1, *gshB* is separated from the main cluster by short predicted open reading frames that may encode proteins of unknown function (Fig. 3A). In both strains, a gene encoding a putative aldehyde dehydrogenase is located between *isoJ* and *isoA*, in common with isoprene degraders *Rhodococcus* strains LB1 and SC4 (El Khawand *et al*., 2016), although additional genes (encoding two hypothetical proteins, an alpha/beta domain‐containing protein, acetyl‐CoA acetyltransferase, and 3‐hydroxyacyl‐CoA dehydrogenase, all of unknown functions) are present in these rhodococci but absent in the *Gordonia* and *Mycobacterium* isolates.

A second SDIMO was identified elsewhere in the genomes of both isolates (Fig. 3B). Based on gene layout and sequence, the enzymes from *Gordonia* sp. i37 and *Mycobacterium* sp. AT1 were predicted to belong to group V of the SDIMO family (Holmes and Coleman, 2008), most similar to propane monoxygenase (PrMO) from *Gordonia* TY5 (Kotani *et al*., 2003) (98–100% amino acid identity) and phenol/propane monooxygenase from *Mycobacterium goodii* sp. 12523 (Furuya *et al*., 2011) (88–97% identity) respectively (Table S5). In *Gordonia* sp. i37, four additional open reading frames downstream of the monooxygenase share an identical layout and high sequence similarity to homologues from *Gordonia* sp. TY5 (Fig. 3B and Supporting Information Table S5), which encode a predicted amidohydrolase, a protein of unknown function, an alcohol dehydrogenase required for propane metabolism and a putative GroEL‐like chaperone (Kotani *et al*., 2003). Homologues of these genes, with the same layout, are also present in *Mycobacterium smegmatis* mc^2^155 and *Mycobacterium goodie* 12523, and the GroEL chaperone was found to be essential for functional expression of the monooxygenase in a heterologous host (Furuya *et al*., 2013). Interestingly, in *Mycobacterium* sp. AT1, the first three of these four genes are in a different chromosomal location and only the GroEL chaperone is immediately downstream of the monooxygenase (Fig. 3B). In addition to isoprene, *Gordonia* sp. i37 and *Mycobacterium* sp. AT1 could grow on propane (although only *Gordonia* sp. i37 could grow on phenol), together with many of the potential intermediates of propane oxidation, as sole carbon source (Table S6). SDIMOs have an extremely wide and well‐documented substrate range (e.g., the soluble methane monooxygenase can co‐oxidise many simple and branched alkanes, alkenes, and aromatic compounds; Colby *et al*., 1977), and we verified that *X. autotrophicus* Py2 can co‐oxidise (but not grow on) isoprene (Table S7), casting doubt on the exclusive roles of these two SDIMOs (IsoMO and PrMO) during growth on isoprene and propane. We therefore investigated the expression and activity of these enzymes during growth on isoprene and propane.

### Transcription and activity of IsoMO and PrMO

**Figure 4:**
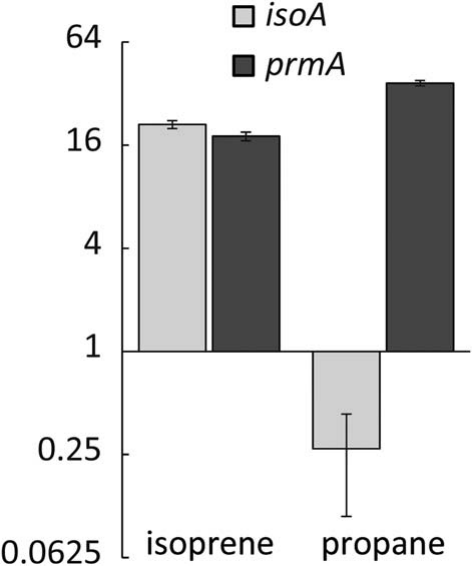
Transcription of *isoA* or *prmA* in cells grown on isoprene or propane. The data show gene transcript abundance in *Gordonia* sp. i37, relative to cells grown on glucose (= 1). Data points represent the mean ± SD, *n* = 3.

Transcription of *isoA* and *prmA*, encoding the oxygenase and hydroxylase alpha subunits of IsoMO and PrMO, respectively, was quantified in *Gordonia* sp. i37 by quantitative reverse transcriptase polymerase chain reaction (RT‐qPCR). Comparison of cells grown on isoprene or propane with those grown on glucose showed that *isoA* was upregulated 21‐fold during growth on isoprene but downregulated fourfold during growth on propane (Fig. 4). However, *prmA*, upregulated 36‐fold in cells grown on propane, was also 18‐fold upregulated in cells grown on isoprene, indicating that growth on isoprene, as well as propane, induced transcription of propane monooxygenase, whereas propane did not induce transcription of IsoMO.

The extent to which gene transcription resulted in the expression of active protein was evaluated by respiratory assays. Oxygen uptake by whole cells of *Gordonia* sp. i37 and *Mycobacterium* sp. AT1, grown on glucose, isoprene or propane, in response to addition of these substrates, was measured polarographically using an oxygen electrode (Table 1). For both isolates, when challenged with propane or isoprene, activity was only detected in cells grown on the corresponding substrate, whereas glucose induced oxygen uptake in cells from any of these three growth conditions. Analysis of the proteins in cell extracts, by 1‐dimensional denaturing polyacrylamide gel electrophoresis (SDS–PAGE), indicated that different polypeptides were present in isoprene‐grown cells than in cells grown on propane (Supporting Information Fig. S4) and analysis by mass spectrometry of bands cut from the gels confirmed the presence of IsoA polypeptides in the extract from isoprene‐grown cells (Table S8). Taken together, these data show that isoprene oxidation is carried out by IsoMO and not PrMO and is an inducible trait and that isoprene‐ and propane‐oxidising abilities are specific to cells grown on the corresponding substrate.

**Table 1:** Oxygen uptake rates [nmol min^−1^ mg^−1^ (dw)] of cells of *Gordonia* sp. i37 and *Mycobacterium* sp. AT1 grown on glucose, isoprene or propane, in response to addition of the same substrates.

Strain	Growth substrate	Oxidation substrate		
		Glucose	Isoprene	Propane
	Glucose	6.5 ± 0.1	BDL	BDL
i37	Isoprene	4.6 ± 0.4	5.8 ± 0.0	BDL
	Propane	4.9 ± 0.0	BDL	3.6 ± 0.2
	Glucose	7.3 ± 0.2	BDL	BDL
AT1	Isoprene	4.5 ± 0.1	5.9 ± 0.1	BDL
	Propane	5.2 ± 0.0	BDL	4.1 ± 0.0

The data show the mean of duplicate samples ± range.

BDL, below detection limit.

## Discussion

This study is the first to use DNA‐SIP to identify active isoprene degraders in the marine environment. We identified Actinobacteria of the genera *Mycobacterium* and *Microbacterium* as the predominant isoprene degraders in DNA‐SIP enrichments. Interestingly, betaproteobacterial isoprene degraders, recently identified in terrestrial soils (El Khawand *et al*., 2016), were not found, suggesting that relatives of these strains able to assimilate isoprene under our experimental conditions were not particularly abundant in this estuarine environment. Although we obtained Gram‐negative isolates (*Loktanella* sp. i8b1 and *Stappia* sp. iL2), labelled sequences of closely related strains were not identified by the DNA‐SIP, implying either that conditions in SIP incubations were not suitable for these strains or, perhaps more likely, that they formed a relatively minor component of the isoprene‐degrading community, but were amenable to laboratory cultivation. Sequencing of *isoA* amplicons, highly specific for isoprene assimilators (El Khawand *et al*., 2016), additionally identified *Rhodococcus*‐like *isoA* sequences as a minor component of the first timepoint. Interestingly, the diversity of isoprene degraders increased over time, suggesting either that *Mycobacterium* was more abundant in the environment, but subsequently outcompeted by strains capable of a higher growth rate, or perhaps that some strains were initially inhibited by the artificially high isoprene concentrations during the enrichments. In addition, we cannot rule out the possibility that some of the strains that became labelled at the later timepoint were crossfeeders, benefitting from labelled carbon released from primary isoprene degraders, although the relatively short incubations (15 days) and the fact that isolates were obtained similar to those labelled, suggests that the enrichment of these species was likely due to primary isoprene consumption. At the second timepoint, our DNA‐SIP data could not confirm *Rhodococcus* and *Gordonia* as isoprene assimilators, although both these taxa were abundant in the enrichments. Closely related strains of *Rhodococcus* differ in terms of their isoprene‐assimilating ability (El Khawand *et al*., 2016) and the data perhaps suggest that both isoprene‐assimilating and non‐isoprene‐assimilating strains (not distinguished by 16S rRNA gene analysis) may have been present in the enrichments.

Phylogenetic analysis of isolates capable of growth on isoprene as sole source of carbon and energy revealed a diverse assortment of strains, mainly Actinobacteria, but also including Gram‐negative members of the Alphaproteobacteria. Several of these, from diverse taxa, had highly similar *isoA* sequences (Fig. 2). The non‐congruent relationship between 16S rRNA gene and *isoA* gene sequences is striking, as is the similarity in sequence and layout of isoprene metabolic genes between the isolates sequenced in this study and previously sequenced isoprene degraders *Rhodococcus* sp. AD45, *Rhodococcus* sp. LB1, *Rhodococcus* sp. SC4 and *Rhodococcus opacus* PD630 (Chen *et al*., 2014; Crombie *et al*., 2015; El Khawand *et al*., 2016), all of which share an identical layout of *isoGHIJ‐aldh1‐isoABCDEF*, with the exception that *Rhodococcus* sp. AD45 lacks the predicted aldehyde dehydrogenase separating *isoJ–isoA* (Fig. 3A).

It is important to note that isoprene‐oxidising ability is not restricted to the IsoMO. For example, both the soluble methane monooxygenase from methanotrophs and alkene monooxygenase from *X. autotrophicus* Py2 can oxidise isoprene at a significant rate (Patel *et al*., 1982) (Table S7), although these organisms cannot grow on isoprene. This raises the possibility that in environments exposed to a mixture of gases, where non‐isoprene‐assimilators are present and also expressing enzymes with isoprene‐oxidising ability (e.g., the soluble methane monooxygenase of methanotrophs growing on methane), co‐metabolism of isoprene may occur, although the inability to further metabolise the toxic epoxide product likely prevents a significant co‐metabolic flux. Interestingly, metabolism of isoprene requires glutathione in *Rhodococcus* sp. AD45 (van Hylckama Vlieg *et al*., 1999), and all sequenced isoprene metabolic gene clusters include glutathione‐*S‐*transferase genes *isoI* and *isoJ* and also glutathione biosynthesis genes *gshA* and *gshB*. Most non‐isoprene‐degrading Gram‐positive bacteria do not use glutathione but instead rely on alternative small thiols, predominately mycothiol in Actinobacteria (Newton *et al*., 1996). Isoprene assimilators, including *Rhodococcus* sp. AD45, *Gordonia* sp. i37 and *Mycobacterium* sp. AT1, contain *gshA* and *gshB* in association with the IsoMO genes, but close homologues are not present in the genomes of related strains *Rhodococcus* sp. RHA1, *Gordonia polyisoprenivorans* or *M. smegmatis* mc^2^155. Since, in *Rhodococcus* sp. AD45, mycothiol was produced in addition to glutathione (Johnson *et al*., 2009), the likelihood is that in these Gram‐positive isoprene assimilators, glutathione is specific to isoprene metabolism. In contrast, characterised straight chain alkene degraders (including propene‐utilizing *X. autotrophicus* Py2, which, while containing a monooxygenase very similar to IsoMO, is incapable of growth on isoprene) conjugate the epoxide product with another small thiol, coenzyme M (Newton *et al*., 1996; Krishnakumar *et al*., 2008), and contain coenzyme M biosynthetic gene clusters (Krum and Ensign, 2000; Mattes *et al*., 2005; Broberg and Clark, 2010), which are absent from the genomes of *Gordonia* sp. i37 and *Mycobacterium* sp. AT1.

The identification of a second SDIMO in each strain, with similarity to characterised propane monooxygenases, highlights that many actinobacterial isoprene degraders are also capable of growth on propane as sole source of carbon and energy (although not the best‐characterised strain, *Rhodococcus* sp. AD45) (Acuña Alvarez *et al*., 2009; Crombie *et al*., 2015; El Khawand *et al*., 2016). It is interesting to note that PrMO‐like gene clusters are present in many diverse non‐isoprene‐degrading Gram‐positive and Gram‐negative strains not known for hydrocarbon degradation, for example, *Streptomyces alni*, *Azoarcus* sp. BH72, *Rhodobacter sphaeroides* and *Bradyrhizobium japonicum* USDA110 (Kaneko *et al*., 2002; Krause *et al*., 2006; Liu *et al*., 2009; Kontur *et al*., 2012). Previous studies have also implicated the PrMO in the oxidation of 2‐propanol or acetone, perhaps suggesting a more general metabolic role for this enzyme, not exclusive to propane oxidation (Kotani *et al*., 2003; Crombie and Murrell, 2014; Furuya *et al*., 2015). The well‐documented wide substrate range of SDIMOs suggested that IsoMO and PrMO might have overlapping roles in the oxidation of isoprene and propane. Although growth on isoprene resulted in appreciable transcription of *prmA*, the oxygen electrode data clearly showed that during growth on either isoprene or propane, metabolic activity of each monooxygenase was specific to its cognate substrate (Fig. 4 and Table 1). These data suggest that the PrMO is also regulated at the post‐transcriptional level, either by repression of translation or by post‐translational control of activity. Examples of post‐transcriptional regulation have been described both in Actinobacteria (Temmerman *et al*., 2000) and in hydrocarbon degradation (Peters *et al*., 2007), and many diverse mechanisms have been identified (Bobrovskyy and Vanderpool, 2013). It is interesting to note that in *Mycobacterium* sp. AT1 and *Gordonia* sp. i37, a putative GroEL‐like chaperone (required for activity of heterologously expressed PrMO of *Mycobacterium*; Furuya *et al*., 2013), is encoded downstream of the PrMO (Fig. 3B). This gene was shown to be transcribed independently of the monooxygenase genes in *Gordonia* TY5 (Kotani *et al*., 2003), suggesting a possible mechanism whereby inactive enzyme might be produced. We previously showed that a downstream intermediate, rather than isoprene itself, was the inducer of *iso* genes in *Rhodococcus* sp. AD45 (Crombie *et al*., 2015) and the precise mechanism by which isoprene and propane metabolism is activated, both in laboratory cultures and in the environment, is the subject of ongoing research in our laboratory.

It would be challenging to conduct these experiments under environmental conditions, and our comparatively high isoprene concentrations may have selected for organisms able to benefit from laboratory conditions. In addition, *isoA* sequences not detected by our primers, or even alternative enzymes and pathways of isoprene metabolism, may exist. In this study, our aim was to expand the diversity of known isoprene degraders and characterise isolates at the genomic and physiological level, which now enables the development of molecular and biochemical methods to investigate isoprene degradation in more environmentally‐relevant conditions. In conclusion, this study is the first to identify and characterise in detail active isoprene degraders in the marine/estuarine environment and significantly advances our understanding of the biodegradation of this environmentally important trace gas.

## Experimental procedures

### Media, enrichment and isolation

Modified MAMS medium was used for the isolation and maintenance of bacterial strains, as described by Schaefer et al. (2002), except that it contained 20 g l^−1^ NaCl and was supplemented with 5 ng l^−1^ of Na_3_VO_4_ and Na_2_SeO_3_. To establish the optimum salinity for the strains, growth was also tested at 14 NaCl concentrations between 0% and 20% (wt/vol) (data not shown and Supporting Information Fig. S3). Isoprene was added to the headspace as vapour (0.5% v/v except where indicated). Alternative gaseous substrates (methane, ethane, propane, butane, propene and 2‐butene) were added to 10% (vol/vol) and other substrates were supplied at 5 mM. For routine growth on isoprene and growth tests on alternative carbon substrates, cultures were incubated at 30°C shaking at 150 rpm in serum vials (120 ml) sealed with grey butyl rubber seals. Aqueous phase isoprene concentrations were estimated based on Henry's law constant *K*_H_ = 1.3 × 10^−2^ (M atm^−1^) (Mackay and Shiu, 1981).

Enrichments for isolation of isoprene‐degrading bacteria contained approximately 100 ml of surface water and 5 g of surface sediment, or water only, for estuarine/coastal or open water sites, respectively, and were incubated with isoprene in the headspace (0.5%, vol/vol) in conical flasks (500 ml) sealed with Suba seals. Isoprene consumption was monitored by gas chromatography as described previously (Crombie *et al*., 2015), and isoprene was replenished when necessary. Turbid cultures were plated onto modified MAMS agar plates and incubated in an atmosphere containing approximately 5% (vol/vol) isoprene. Colonies were transferred back to liquid culture to confirm growth on isoprene. Purity was ensured by streaking to single colonies on minimal and rich media and checked by microscopy.

### DNA stable isotope probing

Unlabelled isoprene was obtained from Sigma Aldrich (Gillingham, UK), and ^13^C‐labelled isoprene was biosynthesised as described previously (El Khawand *et al*., 2016). Water and sediment from Wivenhoe (Colne Estuary, UK) (30 ml water + 1 g sediment) were incubated with 0.2% (vol/vol) ^13^C‐labelled isoprene added to the headspace as sole carbon source in duplicate serum vials (120 ml), together with duplicate controls with unlabelled isoprene. Vials for timepoint two were re‐spiked with isoprene after 12 days. Killed (autoclaved) controls were also included. Since isoprene uptake was initially slow, microcosms were supplemented with a small amount of nutrients in the form of a 1/30 (vol/vol) addition of mineral salts (MAMS) medium at day 7, after which isoprene uptake proceeded rapidly (data not shown). Samples were incubated at room temperature without shaking, and isoprene depletion was measured by gas chromatography as previously described (El Khawand *et al*., 2016). Samples were sacrificed at two timepoints after consumption of approximately 30 and 60 µmol of isoprene C, at 12 and 15 days respectively. Samples were centrifuged at 12,000 × *g* and pellets resuspended in PIPES (piperazine‐*N*‐*N*′‐bis(2‐ethanesulfonic acid)) buffer (100 mM, pH 6.9). DNA was extracted using the FastSpin DNA soil kit (MP Biomedicals, Santa Ana, CA, USA). From each sample, approximately 3 µg of DNA was added to caesium chloride solution for isopycnic ultracentrifugation following the protocol described by Neufeld et al. (2007), to separate ^13^C‐labelled DNA from unlabelled (^12^C) DNA. Each sample was then separated into 12 fractions, density was quantified using a digital refractometer (Reichert AR2000; Reichert Analytical Instruments, Buffalo, NY, USA) and DNA precipitated and re‐suspended according to Neufeld et al. (2007). DNA was evaluated for quality by running on a 1% (wt/vol) agarose gel and quantified using a NanoDrop spectrophotometer. Fractions containing labelled DNA were identified by denaturing gradient gel electrophoresis (DGGE) of 16S rRNA gene amplicons as described previously (El Khawand *et al*., 2016). We used highly conservative criteria to identify labelled OTUs. For both replicates, for each OTU, the ratio of relative abundances in the heavy:light fractions in incubations with labelled substrate should exceed 100:1, but not exceed 2:1 in incubations with unlabelled substrate.

### Amplicon sequencing of 16S rRNA and isoA genes

Labelled and unlabelled DNA from SIP incubations with ^12^C‐ and ^13^C‐isoprene was characterized by the sequencing of 16S rRNA gene amplicons generated by PCR using the primers 27Fmod (5′‐AGRGTTTGATCMTGGCTCAG‐3′) and 519Rmodbio (5′‐GTNTTACNGCGGCKGCTG‐3´) using a Roche 454 FLX titanium instrument at MR DNA (Molecular Research LP, Shallowater, TX, USA). Sequence data were analysed at that facility using a custom pipeline (Dowd *et al*., 2008; Capone *et al*., 2011). Barcodes and primers were removed from Q25 reads, short sequences (< 200 bp), those with ambiguous bases or > 6 bp homopolymer runs were discarded and reads were denoised and chimeric sequences removed. Following clustering, OTUs (defined at 97% sequence identity) were assigned to taxa using Blastn against the RDPII/NCBI database (v 11.1) (Cole *et al*., 2014). The average number of sequences obtained per sample was 5386.

Amplicons generated using *isoA* primers (El Khawand *et al*., 2016) were analysed using Mothur v.1.36.1 (Schloss *et al*., 2009). Short reads were discarded, filtered to a 50 bp quality‐window average of 35 and trimmed to 300 bp, resulting in 1211 reads included in the analysis. Reads were clustered at 97% and representative sequences aligned against *isoA* sequences from known isoprene degraders.

Sequence reads from 16S rRNA and *isoA* gene amplicons have been uploaded to the Sequence Read Archive (SRA) under accession number SRS2045023.

### Sequencing of isolate 16S rRNA and isoA genes

Isolate 16S rRNA and *isoA* genes were amplified using primers 27F/1492R (Lane, 1991) and IsoAF/IsoAR respectively. Amplicons were cloned and sequenced using dideoxy Sanger sequencing as previously described (El Khawand *et al*., 2016). Isolate 16S rRNA gene sequences have been deposited under accession numbers KY649287–KY649296 and *isoA* sequences under accession numbers KU870739–KU870740 and KU870742–KU870746.

### Genome sequencing, annotation and genome mining

The genomes of *Gordonia* sp. i37 and *Mycobacterium* sp. AT1 were sequenced as previously described (El Khawand *et al*., 2016). The sequence data were uploaded to GenBank and annotated by the NCBI Prokaryotic Genome Annotation Pipeline (v4.0). Local nucleotide databases were constructed using NCBI BLAST and searched using tBLASTn with the amino acid sequences of characterised enzymes from *Rhodococcus* sp. AD45 or other alkane or alkene degraders as query sequences. These Whole Genome Shotgun projects have been deposited at DDBJ/EMBL/GenBank under the accession numbers MVOCO1000000 and MVPXO1000000 for *Mycobacterium* sp. AT1 and *Gordonia* sp. i37 respectively.

### Protein analysis

Cells grown on isoprene, glucose, or propane were harvested at OD_540_ 0.8–1.0 by centrifugation (12,000 × *g*, 30 min), washed and resuspended in 50 mM PIPES buffer pH 7.0. Cells were broken by four passages through a French pressure cell (American Instrument Company, Silver Spring, MD, USA) at 110 MPa on ice. Cell debris was removed by centrifugation (10,000 × *g*, 15 min, 4°C), and the supernatant removed as cell‐free extract. Polypeptides were separated using Novex mini gels (ThermoFisher, Waltham, MA, USA) and stained with Coomassie blue.

### Proteomic analysis

Bands of interest were cut from the gels and polypeptides identified by the Biological Mass Spectrometry and Proteomics Group facility (University of Warwick) by tryptic digest and nanoliquid chromatography–electrospray‐ionization mass spectrometry (nano‐LC–ESI‐MS/MS) using a nanoACQUITY/Q‐Tof Ultima Global instrument (Waters, Milford, MA, USA).

### Oxygen electrode assays

Substrate‐induced oxygen consumption was measured using a Clark‐type oxygen electrode (Rank Brothers, Cambridge, UK) maintained at 25°C using a circulating water bath (Churchill, Perivale, UK). Cells were grown and harvested as described above, resuspended in 50 mM phosphate buffer (pH 7.0) containing NaCl (1%, wt/vol) and starved for 30 min on ice. Cell suspension was transferred to the instrument reaction chamber, and the endogenous rate was recorded for 2 min before the addition of substrate (50 µl). Gaseous substrates and isoprene were prepared as saturated solutions in water and other substrates as 100 mM stock solutions. Substrate‐induced oxygen consumption was calculated by the subtraction of endogenous from substrate‐induced rate.

### Oxidation of propene and isoprene by *X. autotrophicus* Py2 and *Rhodococcus* sp. AD45

To demonstrate the substrate versatility of alkene monooxygenase from *X. autotrophicus* Py2 and IsoMO from *Rhodococcus* sp. AD45, cells were grown on propene and isoprene, respectively, as previously described (Small *et al*., 1995; Crombie *et al*., 2015). Cells were harvested by centrifugation (8000 × *g*, 4°C, 20 min), resuspended in phosphate buffer (50 mM, pH 7.0), drop frozen in liquid nitrogen and stored at −80°C. Subsequently, frozen cells were resuspended in buffer to a density of approximately 0.25 mg (dry weight) ml^−1^. Cell suspension (1 ml) was transferred to vials (30 ml), sealed with butyl rubber stoppers, supplied with isoprene vapour or propene (approximately 250 ppmv) and incubated at 30°C with shaking. Substrate depletion was measured by gas chromatography as described previously (Crombie *et al*., 2015).

### RNA extraction and RT‐qPCR

Total RNA was extracted using the acid‐phenol method of Gilbert *et al*., (2000). Residual DNA was removed by two treatments with RNase‐free DNase (Qiagen, Crawley, UK) following the manufacturer's instructions and confirmed by 16S rRNA PCR using an RNA template. cDNA was synthesised using Superscript III (Invitrogen), according to the manufacturer's instructions, using random hexamers and 50–1000 ng RNA. Negative controls contained water in place of reverse transcriptase. Quantitative PCR was conducted using the Applied Biosystems SYBR Green Master Mix and a StepOnePlus instrument (ThermoFisher). Target copy number was quantified against a dilution series of standards for each target gene (included in every plate) and *isoA* or *prmA* gene expression in isoprene‐ and propane‐grown cells, normalized to *rpoB* as reference, was reported as fold‐change in comparison to glucose‐grown cells.

## Supporting information

Additional Supporting Information may be found in the online version of this article at the publisher's web‐site:

**Table S1.** Isolate 16S rRNA gene‐based taxonomic affiliation, origin and growth characteristics in batch culture with isoprene as sole source of carbon and energy.
**Table S2.** Basic genome data for *Gordonia* sp. i37 and *Mycobacterium* sp. AT1.
**Table S3.** Additional predicted short chain alkane or alkene oxidising genes.
**Table S4.** Predicted functions of isoprene‐related genes in *Gordonia* sp. i37 (i37) and *Mycobacterium* sp. AT1 (AT1). The table shows the amino acid similarity (% identity) of the sequences from each strain compared with *Rhodococcus* sp. AD45 (AD45) and also with each other.
**Table S5.** Amino acid similarity (% identity) of the propane monooxygenases and associated predicted gene products of *Gordonia* sp. i37 (i37) and *Mycobacterium* sp. AT1 (AT1) with the characterised enzymes of *Gordonia* sp. TY5 (TY5) (Kotani et al., 2003) and *Mycobacterium goodii* sp. 12523 (12523) or *Mycobacterium smegmatis* mc^2^155 (mc^2^155) (where sequence data for *M. goodii* are not available) (Furuya et al., 2011) respectively.
**Table S6.** Growth of isoprene‐degrading isolates on selected alternative substrates.
**Table S7.** Oxidation of propene and isoprene by cell suspensions of *Xanthobacter autotrophicus* Py2 and *Rhodococcus* sp. AD45. Data show the mean of 4 replicates ± SD (except *X. autotrophicus* Py2, propene, three replicates).
**Table S8.** Isoprene monooxygenase IsoA polypeptides detected in bands cut from SDS–PAGE gels loaded with cell extract from *Gordonia* sp. i37 (Supporting Information Fig. S4) and *Mycobacterium* sp. AT1, grown on isoprene.
**Fig. S1.** Genus‐level unenriched and enriched communities generated by DNA‐SIP, analysed by 16S rRNA gene amplicon sequencing. The unenriched timepoint zero community is shown together with enriched communities at timepoints one and two (12 and 15 days), retrieved from the heavy and light fractions of ^13^C‐ and ^12^C‐isoprene incubations. All taxa present with a relative abundance > 1% at any timepoint are shown. Sequences retrieved from heavy or light fractions of each replicate (1 or 2) are prefixed H or L. The communities labelled by incubation with ^13^C isoprene are outlined with dashed rectangles.
**Fig. S2.** Phylogenetic tree showing relationship of operational taxonomic units (OTUs) generated by 454 sequencing of *isoA* amplicons from DNA‐SIP labelled fractions of timepoint 1 (12 days). The tree was constructed using the Maximum Likelihood method in MEGA6 (Tamura *et al*., 2013). Gaps and missing data were removed and there were 297 nucleotide positions in the final dataset. The scale bar shows nucleotide substitutions per site. Bootstrap values (1000 replications) are shown at the nodes. DNA‐SIP amplicons are indicated with the prefix OTU and show relative abundance in parentheses. OTUs present at less than 0.5% relative abundance are not shown.
**Fig. S3.** Growth of *Gordonia* sp. i37 (left) and *Mycobacterium* sp. AT1 (right) on isoprene (approximately 1%, vol/vol, in the headspace) at salinities between 0% and 5% (wt/vol).
**Fig. S4.** Protein extracted from cells grown on succinate (S), propane (P) or isoprene (I), separated by SDS–PAGE. A band, corresponding to that indicated with an arrow, was cut from a lane of a similar gel loaded with isoprene‐grown extract, for mass spectrometry analysis, indicating the presence of oxygenase alpha‐subunit (IsoA) peptides (Table S8). M, molecular mass marker.
