# Supplementary material for: Identification and characterisation of isoprene‐degrading bacteria in an estuarine environment
Source: Environ Microbiol. 2017 Jul 21;19(9):3526–37. doi: 10.1111/1462-2920.13842 (PMC6849523; doi:10.1111/1462-2920.13842)
Supplement: Supplementary file 1 — Table S1. Isolate 16S rRNA gene‐based taxonomic affiliation, origin and growth characteristics in batch culture with isoprene as sole source of carbon and energy. Table S2. Basic genome data for Gordonia sp. i37 and Mycobacterium sp. AT1. Table S3. Additional predicted short chain alkane or alkene oxidising genes. Table S4. Predicted functions of isoprene‐related genes in Gordonia sp. i37 (i37) and Mycobacterium sp. AT1 (AT1). The table shows the amino acid similarity (% identity) of the sequences from each strain compared with Rhodococcus sp. AD45 (AD45) and also with each other. Table S5. Amino acid similarity (% identity) of the propane monooxygenases and associated predicted gene products of Gordonia sp. i37 (i37) and Mycobacterium sp. AT1 (AT1) with the characterised enzymes of Gordonia sp. TY5 (TY5) (Kotani et al., 2003) and Mycobacterium goodii sp. 12523 (12523) or Mycobacterium smegmatis mc2155 (mc2155) (where sequence data for M. goodii are not available) (Furuya et al., 2011) respectively. Table S6. Growth of isoprene‐degrading isolates on selected alternative substrates. Table S7. Oxidation of propene and isoprene by cell suspensions of Xanthobacter autotrophicus Py2 and Rhodococcus sp. AD45. Data show the mean of 4 replicates ± SD (except X. autotrophicus Py2, propene, three replicates). Table S8. Isoprene monooxygenase IsoA polypeptides detected in bands cut from SDS–PAGE gels loaded with cell extract from Gordonia sp. i37 (Supporting Information Fig. S4) and Mycobacterium sp. AT1, grown on isoprene. Fig. S1. Genus‐level unenriched and enriched communities generated by DNA‐SIP, analysed by 16S rRNA gene amplicon sequencing. The unenriched timepoint zero community is shown together with enriched communities at timepoints one and two (12 and 15 days), retrieved from the heavy and light fractions of 13C‐ and 12C‐isoprene incubations. All taxa present with a relative abundance > 1% at any timepoint are shown. Sequences retrieved from heavy or light fraction [file EMI-19-3526-s001.docx]

**Table S1.** Isolate 16S rRNA gene-based taxonomic affiliation, origin and growth characteristics in batch culture with isoprene as sole source of carbon and energy.

| Isolate | Representative NCBI blast hit (% identity) | Specific growth rate (h^-1^) | Doubling time (h) | Culture density obtained (OD_540_) | Reference (sampling location) |
| --- | --- | --- | --- | --- | --- |
| *Gordonia* sp. i37 | *Gordonia polyisoprenivorans* VH2 (99) | 0.16 | 4.2 | 0.88 | Acuña Alvarez et al., 2009 |
| *Leifsonia* sp. i49^1^ | *Leifsonia xyli* HS0904 (100) | 0.02 | 44.0 | 0.12 | Acuña Alvarez et al., 2009 |
| *Loktanella* sp. i8b1 | *Loktanella salsilacus* KMGL1309-SO6 (99) | 0.07 | 9.9 | 0.54 | This study  (Colne estuary) |
| *Micrococcus* sp. i61b | *Micrococcus luteus* VN2013-69 (100) | 0.12 | 5.8 | 0.67 | This study  (Colne estuary) |
| *Mycobacterium* sp. AT1 | *Mycobacterium rhodesiae* NBB3 (98) | 0.06 | 11.0 | 1.14 | Acuña Alvarez et al., 2009 |
| *Mycobacterium* sp. i61a | *Mycobacterium parafortuitum* 161-3 (99) | 0.08 | 8.1 | 0.30 | This study  (Colne estuary) |
| *Rhodococcus* sp. i29a2 | *Rhodococcus erythropolis* (99) | 0.04 | 19.7 | 0.69 | This study  (Colne estuary) |
| *Rhodococcus* sp. i47 | *Rhodococcus opacus* 04-OD7 (100) | 0.13 | 5.4 | 0.72 | This study  (Colne estuary) |
| *Rhodococcus* sp. i8a2 | *Rhodococcus.globerulus* NCIMB 12315T (100) | 0.04 | 15.5 | 0.73 | This study  (Colne estuary) |
| *Stappia* sp. iL2 | *Stappia indica* B106 (99) | 0.04 | 18.0 | 0.35 | This study  (Station L4, WCO) |

^1^ This strain was originally designated *Leifsonia* Strain i47. WCO, Western Channel Observatory.

**Table S2.** Basic genome data for *Gordonia* sp. i37 and *Mycobacterium* sp. AT1

| **Strain** | **Size (Mbp)** | **# contigs** | **GC content (%)** | **CDS** | **tRNAs** |
| --- | --- | --- | --- | --- | --- |
| *Gordonia* sp. i37 | 6.23 | 721 | 66.8 | 5,909 | 48 |
| *Mycobacterium* sp. AT1 | 7.07 | 129 | 67.2 | 6,758 | 45 |

**Table S3**. Additional predicted short chain alkane or alkene oxidising genes

| Locus tag (accession no.) | Predicted function | Characterised enzyme (% amino acid identity) | NCBI hit (% amino acid identity) | Comments |
| --- | --- | --- | --- | --- |
| *Gordonia* sp. i37 |  |  |  |  |
| B1964_20110 (OPX13471.1) | Cytochrome CYP153 | CYP153A16 *Mycobacterium marinum*^1^ ACC41588.1 (68%) | Cytochrome P450,  *Gordonia polyisoprenivorans* WP_014361693.1 (97%) | Fusion with ferredoxin and ferredoxin reductase domains |
| B1964_28090 (OPX07206.1) | Reductase |  | Ferredoxin reductase, *Gordonia amicalis* WP_006437591.1 (99%) |  |
| B1964_28095 (OPX07207.1) | Cytochrome CYP153 | CYP153A16 *Mycobacterium marinum*^1^ ACC41588.1 (81%) | Cytochrome P450,  *Gordonia polyisoprenivorans* NBRC 16320 GAB22983.1 (100%) |  |
| B1964_28100 (OPX07208.1) | Ferredoxin |  | 2Fe-2S ferredoxin, *Gordonia polyisoprenivorans* NBRC 16320 (100%) |  |
|  |  |  |  |  |
| *Mycobacterium* sp. AT1 | |  |  |  |
| B1790_12785 (OPX10085.1) | Rubredoxin |  | Rubredoxin, *Mycobacterium rhodesiae* WP_014208871.1 (84%) |  |
| B1790_12790 (OPX10086.1) | Rubredoxin |  | Alkane 1-monooxygenase, *Mycobacterium* sp. Root135 KQY04364.1 (93%) |  |
| B1790_12795 (OPX10087.1) | Alkane 1-MO | AlkB *Mycobacterium tuberculosis* H37Rv^2^ O05895 (81%) | Rubredoxin, *Mycobacterium* sp. Root135 WP_056556065.1 (98%) |  |
| B1790_19650 (OPX08324.1) | Cytochrome CYP153 | CYP153A16 *Mycobacterium marinum*^1^ ACC41588.1 (85%) | Cytochrome P450, *Mycobacterium* sp. Root135 WP_056547397.1 (98%) |  |

^1^Scheps et al., (2011) ^2^Smits et al., (2002)

**Table S4.** Predicted functions of isoprene-related genes in *Gordonia* sp. i37 (i37) and *Mycobacterium* sp. AT1 (AT1). The table shows the amino acid similarity (% identity) of the sequences from each strain compared with *Rhodococcus* sp. AD45 (AD45) and also with each other.

| **Gene** | **Predicted function** | **i37 : AD45** | **AT1 : AD45** | **i37 : AT1** |
| --- | --- | --- | --- | --- |
| *gshA* | Glutamate-cysteine ligase | 55 | 57 | 55 |
| *isoG* | Racemase | 79 | 84 | 82 |
| *isoH* | HGMB dehydrogenase | 79 | 78 | 82 |
| *isoI* | Glutathione-*S*-transferase | 80 | 82 | 83 |
| *isoJ* | Glutathione-*S*-transferase | 71 | 74 | 70 |
| *aldh1* | Aldehyde dehydrogenase | 74 | 77 | 78 |
| *isoA* | IsoMO, α-subunit | 86 | 86 | 87 |
| *isoB* | IsoMO, γ-subunit | 57 | 72 | 66 |
| *isoC* | IsoMO, ferredoxin | 78 | 82 | 80 |
| *isoD* | IsoMO, coupling protein | 71 | 74 | 73 |
| *isoE* | IsoMO, β-subunit | 71 | 65 | 72 |
| *isoF* | IsoMO, reductase | 60 | 57 | 58 |
| *aldh2* | Aldehyde dehydrogenase | 53 | 57 | 56 |
| *gshB* | Glutathione synthetase | 65 | 67 | 66 |
| - | CoA-DSR | 62 | 61 | 66 |

**Table S5.** Amino acid similarity (% identity) of the propane monooxygenases and associated predicted gene products of *Gordonia* sp. i37 (i37) and *Mycobacterium* sp. AT1 (AT1) with the characterised enzymes of *Gordonia* sp. TY5 (TY5) (Kotani et al., 2003) and *Mycobacterium* *goodii* sp. 12523 (12523) or *Mycobacterium smegmatis* mc^2^155 (mc^2^155) (where sequence data for *M. goodii* are not available) (Furuya et al., 2011), respectively.

| **gene** | **Description** | **i37 : TY5** | **AT1 : 12523** |
| --- | --- | --- | --- |
| *prmA* | Hydroxylase α-subunit | 99 | 97 |
| *prmB* | Reductase | 91 | 88 |
| *prmC* | Hydroxylase β-subunit | 98 | 92 |
| *prmD* | Coupling protein | 100 | 94 |
|  |  | **i37 : TY5** | **AT1 : mc^2^155** |
| *orf1* | Amidohydrolase | 99 | 71* |
| *orf2* | Unknown | 99 | 60* |
| *adh1* | Alcohol dehydrogenase | 100 | 90* |
| *orf3* | GroEL chaperone | 88 | 87 |

*In *Mycobacterium* sp. AT1, *orf1, orf2* and *adh1* are located on contig 79 whereas the PrMO and GroEL genes are on contig 84.

**Table S6.** Growth of isoprene-degrading isolates on selected alternative substrates.


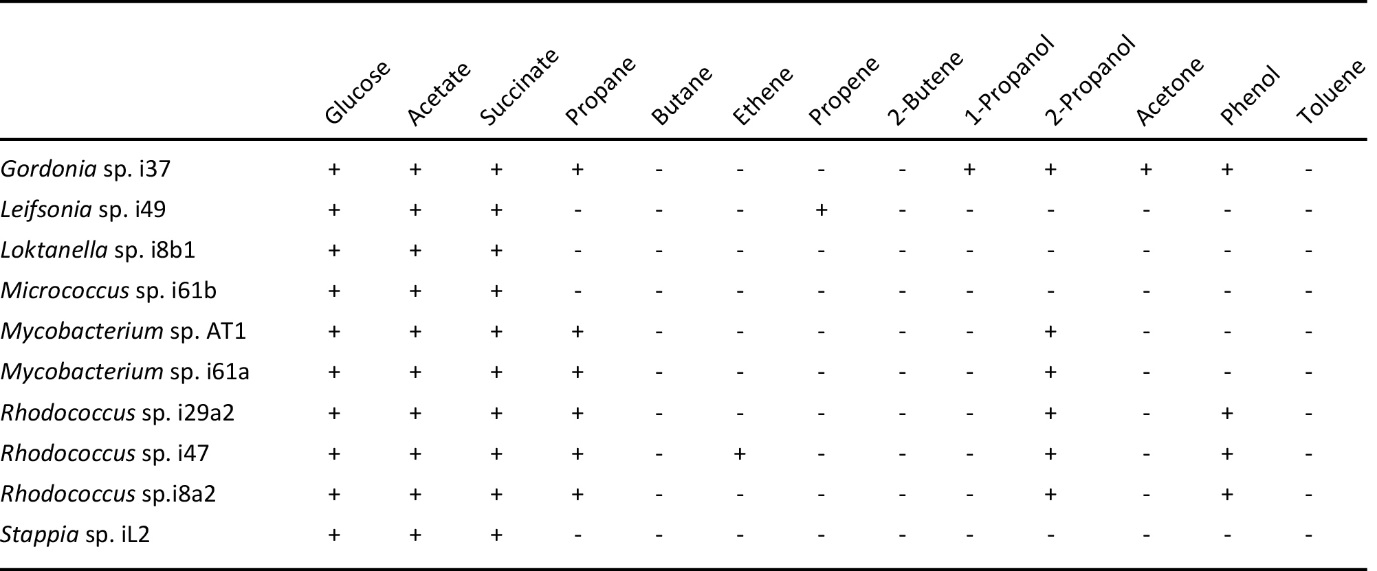


**Table S7.** Oxidation of propene and isoprene by cell suspensions of *Xanthobacter autotrophicus* Py2 and *Rhodococcus* sp. AD45. Data show the mean of 4 replicates ± s.d. (except *X. autotrophicus* Py2, propene, 3 replicates)

|  | **Oxidation rate (nmol min^-1^ mg (dw)^-1^** | |
| --- | --- | --- |
|  | **Propene** | **Isoprene** |
| *Xanthobacter autotrophicus* Py2 | 8.1 ± 1.2 | 3.4 ± 1.0 |
| *Rhocococcus* sp. AD45 | 3.2 ± 0.6 | 9.7 ± 1.9 |

**Table S8.** Isoprene monooxygenase IsoA polypeptides detected in bands cut from SDS-PAGE gels loaded with cell extract from *Gordonia* sp. i37 (Figure S4) and *Mycobacterium* sp. AT1, grown on isoprene

| **Strain** | **Theoretical MM (Da)** | **Amino acids** | **Peptides detected** | **Coverage (%)** |
| --- | --- | --- | --- | --- |
| *Gordonia* sp. i37 | 58,256 | 506 | 5 | 9.49 |
| *Mycobacterium* sp. AT1 | 58,634 | 511 | 2 | 5.25 |


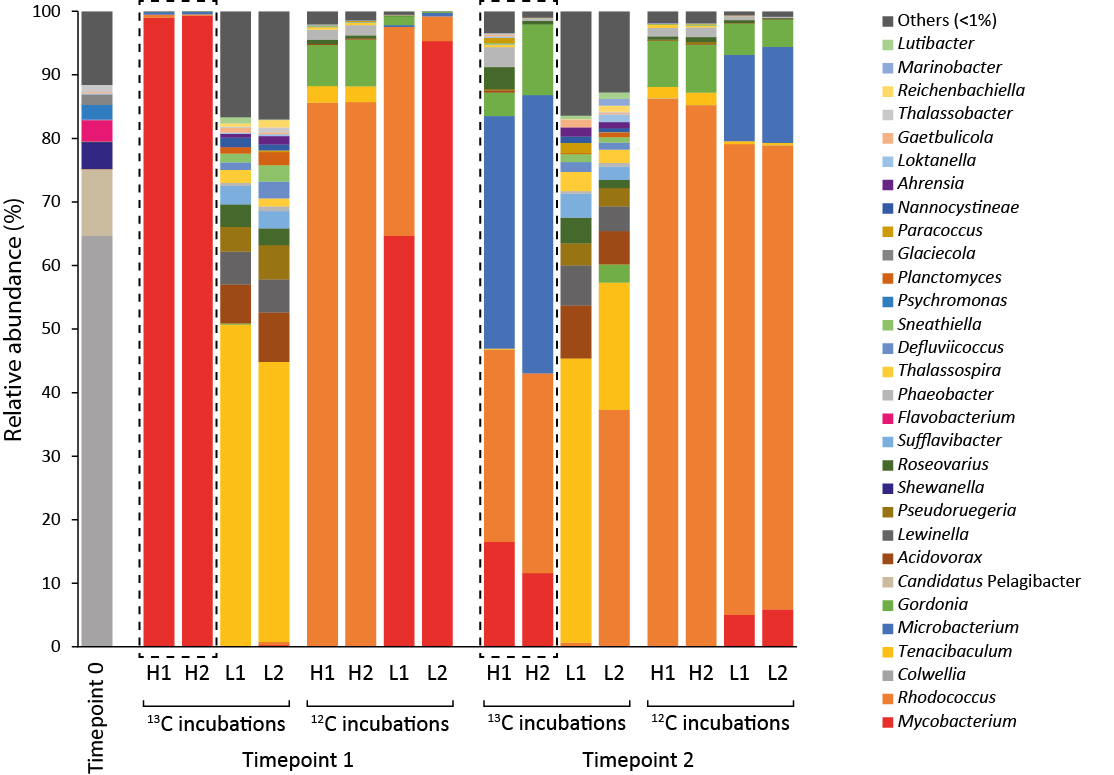


**Figure S1**. Genus-level un-enriched and enriched communities generated by DNA-SIP, analysed by 16S rRNA gene amplicon sequencing. The unenriched timepoint zero community is shown together with enriched communities at timepoints one and two (12 and 15 days), retrieved from the heavy and light fractions of ^13^C- and ^12^C-isoprene incubations. All taxa present with a relative abundance > 1% at any timepoint are shown. Sequences retrieved from heavy or light fractions of each replicate (1 or 2) are prefixed H or L. The communities labelled by incubation with ^13^C isoprene are outlined with dashed rectangles.


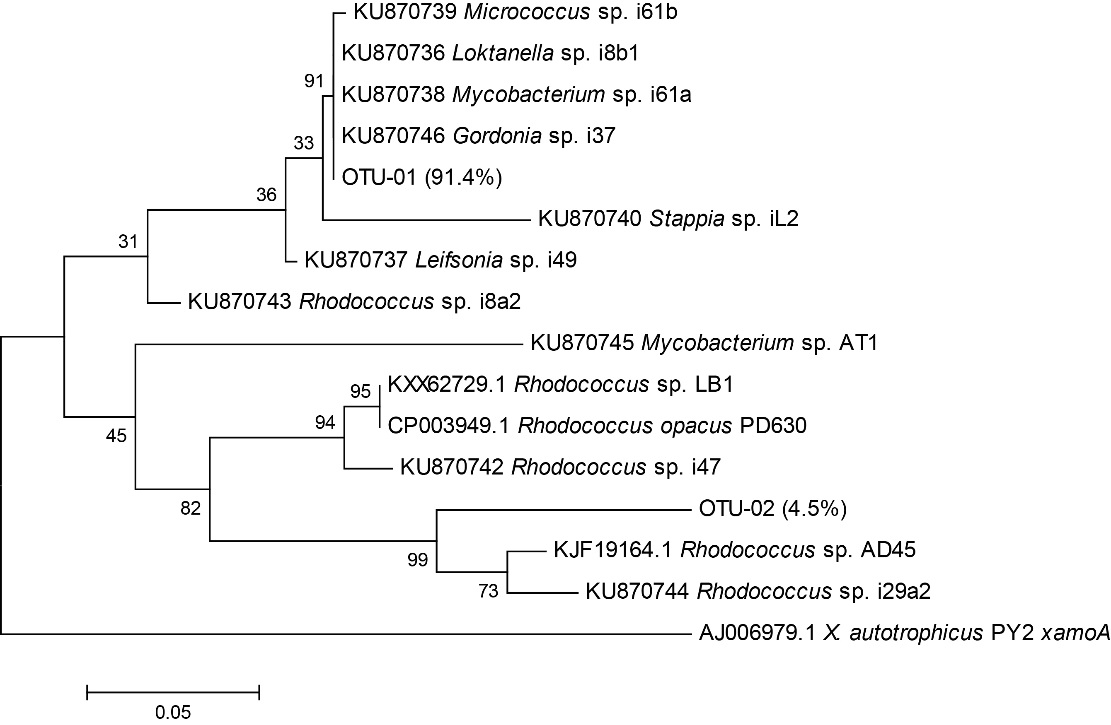


**Figure S2**. Phylogenetic tree showing relationship of operational taxonomic units (OTUs) generated by 454 sequencing of *isoA* amplicons from DNA-SIP labelled fractions of timepoint 1 (12 days). The tree was constructed using the Maximum Likelihood method in MEGA6 (Tamura et al., 2013). Gaps and missing data were removed and there were 297 nucleotide positions in the final dataset. The scale bar shows nucleotide substitutions per site. Bootstrap values (1,000 replications) are shown at the nodes. DNA-SIP amplicons are indicated with the prefix OTU and show relative abundance in parentheses. OTUs present at less than 0.5% relative abundance are not shown.


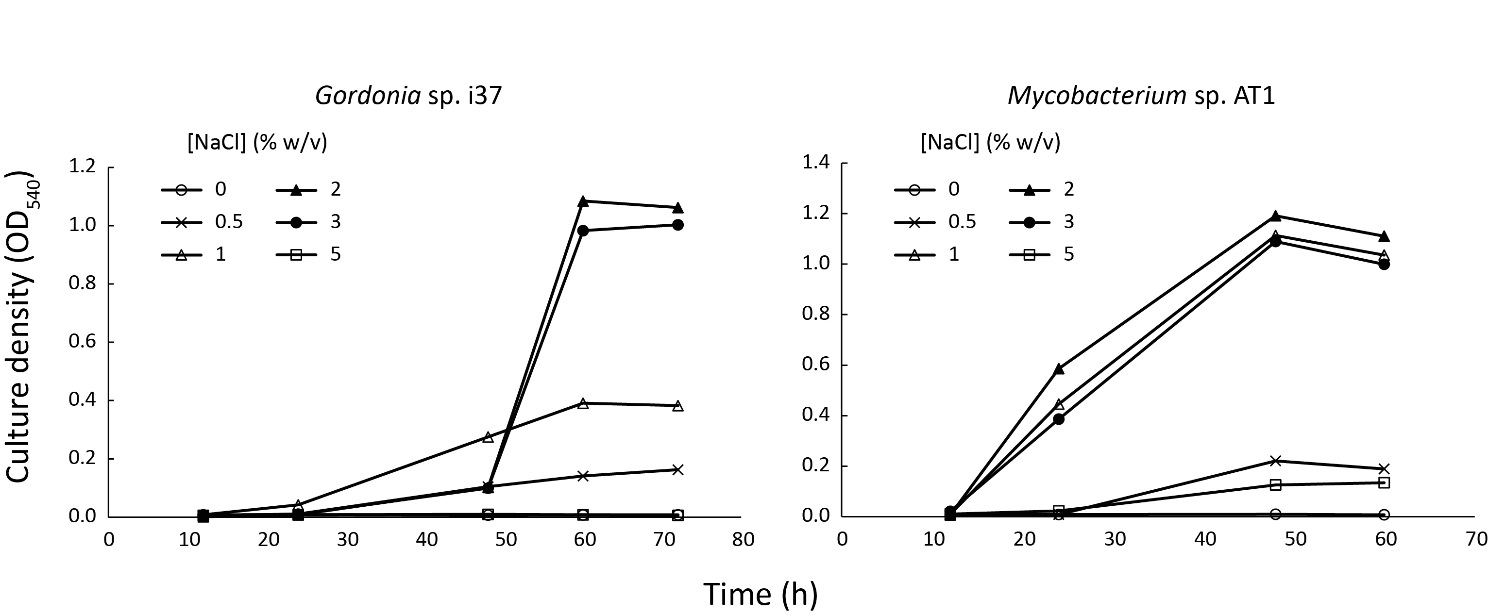


**Figure S3.** Growth of Gordonia sp. i37 (left) and Mycobacterium sp. AT1 (right) on isoprene (approx. 1% v/v in the headspace) at salinities between 0 – 5% (w/v).


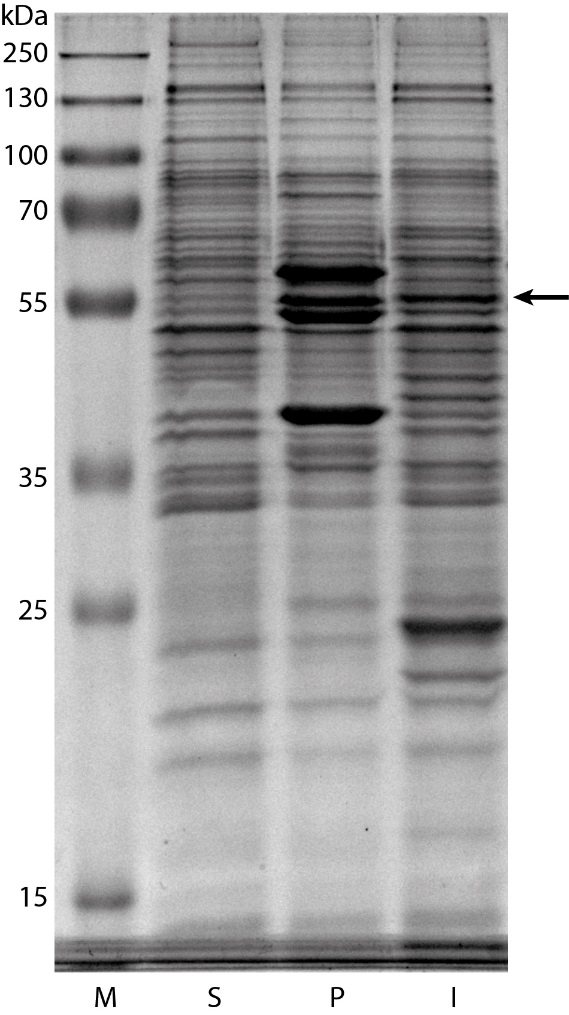


**Figure S4.** Protein extracted from cells grown on succinate (S), propane (P), or isoprene (I), separated by SDS-PAGE. A band, corresponding to that indicated with an arrow, was cut from a lane of a similar gel loaded with isoprene-grown extract, for mass spectrometry analysis, indicating the presence of IsoMO oxygenase alpha-subunit (IsoA) peptides (Table S8). M, molecular mass marker.

**References**

Acuña Alvarez, L., Exton, D.A., Timmis, K.N., Suggett, D.J., and McGenity, T.J. (2009) Characterization of marine isoprene-degrading communities. *Environ Microbiol* **11**: 3280-3291.

Furuya, T., Hirose, S., Osanai, H., Semba, H., and Kino, K. (2011) Identification of the monooxygenase gene clusters responsible for the regioselective oxidation of phenol to hydroquinone in mycobacteria. *Appl Environ Microbiol* **77**: 1214-1220.

Kotani, T., Yamamoto, T., Yurimoto, H., Sakai, Y., and Kato, N. (2003) Propane monooxygenase and NAD^+^-dependent secondary alcohol dehydrogenase in propane metabolism by *Gordonia* sp. strain TY-5. *J Bacteriol* **185**: 7120-7128.

Scheps, D., Honda Malca, S., Hoffmann, H., Nestl, B.M., and Hauer, B. (2011) Regioselective ω-hydroxylation of medium-chain *n*-alkanes and primary alcohols by CYP153 enzymes from *Mycobacterium marinum* and *Polaromonas* sp. strain JS666. *Organic & Biomolecular Chemistry* **9**: 6727-6733.

Smits, T.H.M., Balada, S.B., Witholt, B., and van Beilen, J.B. (2002) Functional analysis of alkane hydroxylases from Gram-negative and Gram-positive bacteria. *J Bacteriol* **184**: 1733-1742.

Tamura, K., Stecher, G., Peterson, D., Filipski, A., and Kumar, S. (2013) MEGA6: Molecular Evolutionary Genetics Analysis version 6.0. *Mol Biol Evol* **30**: 2725-2729.
